# New Alternately Colored FRET Sensors for Simultaneous Monitoring of Zn^2+^ in Multiple Cellular Locations

**DOI:** 10.1371/journal.pone.0049371

**Published:** 2012-11-16

**Authors:** Jose G. Miranda, Amanda L. Weaver, Yan Qin, J. Genevieve Park, Caitlin I. Stoddard, Michael Z. Lin, Amy E. Palmer

**Affiliations:** 1 Department of Chemistry and Biochemistry and BioFrontiers Institute, University of Colorado, Boulder, Colorado, United States of America; 2 Department of Pediatrics and Engineering, Stanford Medical School, Stanford, California, United States of Americs; UMCG, The Netherlands

## Abstract

Genetically encoded sensors based on fluorescence resonance energy transfer (FRET) are powerful tools for reporting on ions, molecules and biochemical reactions in living cells. Here we describe the development of new sensors for Zn^2+^based on alternate FRET-pairs that do not involve the traditional CFP and YFP. Zn^2+^ is an essential micronutrient and plays fundamental roles in cell biology. Consequently there is a pressing need for robust sensors to monitor Zn^2+^ levels and dynamics in cells with high spatial and temporal resolution. Here we develop a suite of sensors using alternate FRET pairs, including tSapphire/TagRFP, tSapphire/mKO, Clover/mRuby2, mOrange2/mCherry, and mOrange2/mKATE. These sensors were targeted to both the nucleus and cytosol and characterized and validated in living cells. Sensors based on the new FRET pair Clover/mRuby2 displayed a higher dynamic range and better signal-to-noise ratio than the remaining sensors tested and were optimal for monitoring changes in cytosolic and nuclear Zn^2+^. Using a green-red sensor targeted to the nucleus and cyan-yellow sensor targeted to either the ER, Golgi, or mitochondria, we were able to monitor Zn^2+^ uptake simultaneously in two compartments, revealing that nuclear Zn^2+^ rises quickly, whereas the ER, Golgi, and mitochondria all sequester Zn^2+^ more slowly and with a delay of 600–700 sec. Lastly, these studies provide the first glimpse of nuclear Zn^2+^ and reveal that nuclear Zn^2+^ is buffered at a higher level than cytosolic Zn^2+^.

## Introduction

Fluorescent proteins (FPs) are powerful tools to monitor cellular signals. Since the initial development of GFP as a research tool for biological discovery, laboratories have diversified FP spectra through directed evolution, resulting in a plethora of probes across the visible spectrum [1]. These FPs have been used in the generation of fluorescence resonance energy transfer (FRET)-based sensors to report dynamic biochemistry in living cells [2], [3]. Because FRET efficiency is sensitive to distance and orientation between the donor and acceptor fluorophore, conformational changes due to binding of a ligand to a protein of interest can form the basis of FRET-based biosensors. The most commonly used donor and acceptor FPs are variants of cyan FP (CFP) and yellow FP (YFP) [3].

In recent years the development of alternate color FRET sensors has enabled new avenues of research such as the ability to monitor a single signal in multiple cellular compartments or simultaneously track two cellular signals [4]. For example, two complementary probes for caspase-3 activity based on mTFP1/mCitrine and mAmetrine/tdTomato were used to visualize caspase-3 activity in the nucleus and cytoplasm, revealing temporal differences in caspase-3 activation [5]. The same FRET pairs were used to develop probes for monitoring both Ca^2+^ and caspase-3 in the same cell [6]. Monomeric Teal FP (mTFP) is a FP version of the widely used CFP derived as a replacement for enhanced CFP because of its high quantum yield [7]. Such studies allow researchers to precisely correlate the timing of two interdependent cellular events or to track the movement of ions or molecules from one compartment to another. An additional advantage of alternate color FRET sensors, particularly those that avoid using a variant of YFP which is quenched by acid [8], is that they are likely to be less sensitive to pH perturbations.

While in principle the concept of generating alternate color FRET sensors is attractive, in practice there are a number challenges that have limited availability of non-CFP/YFP biosensors. First and foremost, the vast majority of the>120 FRET-based biosensors currently available are based on CFP/YFP and as noted in a recent publication [6], changing the FPs often requires extensive re-optimization of the sensor. Secondly, the biophysical (folding, maturation, oligomerization state) and photophysical properties (brightness) of red and orange FPs still lag behind those of the cyan-yellow counterparts [9], making it challenging to identify a robust alternate FRET pair. Indeed of the non-CFP/YFP biosensors developed thus far, each research team chose a different combination of FRET partners [5], [10], [11], [12], [13], [14].

In this work, we developed alternately colored Zn^2+^ biosensors, testing a series of green-red and orange-red FP combinations. Because it is common for sensors to exhibit diminished responses in cells compared to in vitro [15], [16], we screened the panel of sensors in mammalian cells to assess whether they were capable to responding to manipulation of cellular Zn^2+^ levels. The sensors were then targeted to both the nucleus and cytosol and nuclear sensors were used in conjunction with an organelle-localized CFP-YFP-based Zn^2+^ sensor to monitor Zn^2+^ fluxes in two cellular compartments simultaneously. We believe these represent an important breakthrough in expanding the palette of Zn^2+^ sensors.

**Figure 1:**
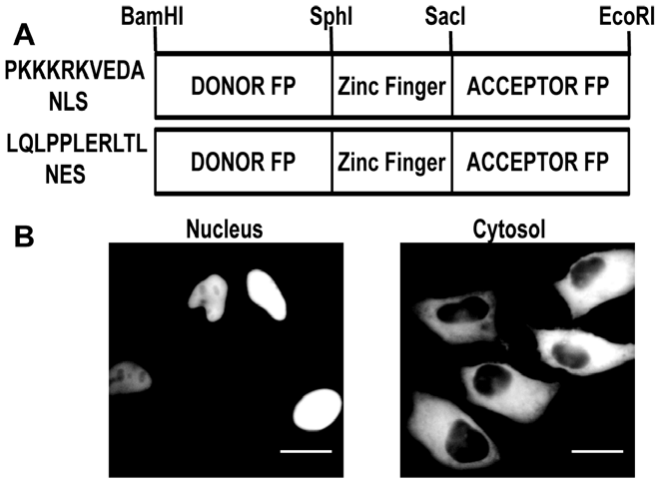
Nuclear Localization and Nuclear Exclusion Signal Sequence constructs. A NLS and NES were cloned into pcDNA 3.1 (+) vector upstream *Bam*H I. A) Schematic of FRET sensor construct. B) Representative images of transfected sensor showing localization to either the nucleus or cytosol. Scale bar = 20 µm.

## Methods

### FRET Sensor Cloning

A schematic of the general sensor construction is presented in **Figure 1A** and all sequences have been deposited in GenBank. **Table S1** summarizes the mutations in the zinc finger domain. For bacterial expression, sensor cDNA was cloned into pET302/NT-His (Life Technologies) in which the *Bam*HI and *Eco*RI restriction sites were reversed. For mammalian cell expression, sensor cDNA was cloned into pcDNA3.1(+) between *Bam*HI and *Eco*RI. To localize sensors to either the nucleus or the cytosol, a nuclear localization (NLS) or nuclear exclusion (NES) signal sequence was cloned upstream of the *Bam*HI site, such that the signal sequence is at the N-terminus of the sensor. For nuclear or cytosolic localization the following primers were used: 5′-**ATG**CCTAAAAAAAAACGTAAAGTTGAAGATGCT**GGATCC**-3′ (NLS) and 5′-**ATG**CTTCAACTTCCTCCTCTTGAACGTCTTACTCTT**GGATCC**-3′ (NES). Sensors containing localization sequences for endoplasmic reticulum, Golgi apparatus, and mitochondria were developed previously [15], [17].

**Table 1:** Fluorescent Protein Excitation and Emission.

Donor FP	Acceptor FP	Sensor Name	Excitation max (nm)	Emission max (nm)
**C**FP	**Y**FP	Zap**CY**2	435	535
t**S**apphire	**m**KO	Zap**SM**2	399	559
t**S**apphire	Tag**R**FP	Zap**SR**2	399	580
m**O**range2	m**C**herry	Zap**OC**2	549	610
m**O**range2	m**K** ATE	Zap**OK**2	549	633
**C**lover	**mR**uby2	Zap**CmR**1	486	605
**C**lover	**mR**uby2	Zap**CmR**1.1	486	605
**C**lover	**mR**uby2	Zap**CmR**2	486	605

Senor nomenclature is as follows: Zap refers to the 1^st^ two zinc fingers of the *Saccaromyces cerevisiae* Zap1 transcription factor that serves as the zinc binding domain, the next two letters refer to the donor and acceptor FPs, finally the “1” at the end of a sensor name indicates the wild type Zap1 domain was used, the “2” indicates that two mutations (Cys to His) were incorporated to lower the zinc affinity, as outlined in [15]. “1.1” indicates one mutation (Cys to His) was incorporated.

Clover lacks the C-terminal residues GITLMDELYK that are present in other GFP-based proteins. During the initial cloning of ZapCmR1 there was an inadvertent addition of the linker MVSKGEEL to the N-terminus of mRuby2 so the sensor contains this additional linker.

**Figure 2:**
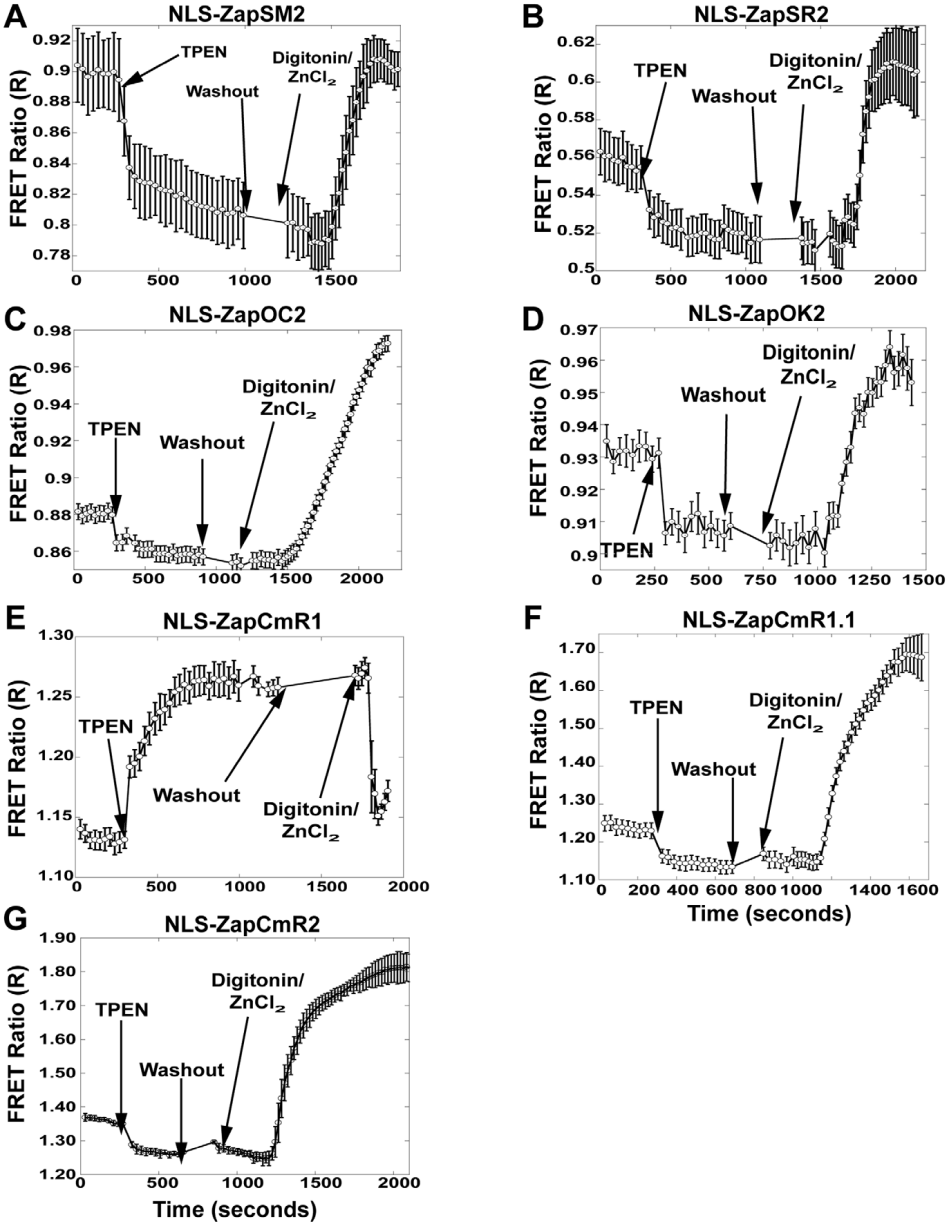
FRET Sensor calibration in the nucleus. Representative calibrations of each sensor localized to the nucleus. The background corrected FRET ratio (FRET Intensity ÷ Donor Intensity) is represented as a function of time. Calibrations were performed by adding 150 µM TPEN to achieve R_TPEN_, followed by washing of residual TPEN and addition of 135 µM ZnCl_2_ with 10 µM Digitonin to permeabilize the cell membrane and obtain R_Zn_. A) NLS-ZapSM2 FRET ratio increases slightly above resting suggesting that it is close to saturation at rest; B) NLS-ZapSR2, FRET ratio goes above resting; C) NLS-ZapOC2 has a small decrease in FRET ratio after TPEN and a larger increase after treatment with Zn^2+^; D) NLS-ZapOK2 exhibits a small change in FRET ratio after TPEN and Zn^2+^; E) NLS-ZapCmR1 has an inverted response in which TPEN causes an increase in FRET ratio while Zn^2+^ with digitonin causes a decrease in the ratio; F) NLS-ZapCmR1.1 displays a decrease in the FRET ratio after TPEN and large increase with Zn^2+^ and digitonin; G) NLS-ZapCmR2 is similar to ZapCmR1.1. Representative traces are mean ± s.e.m. (n = 4 cells). Each experiment was repeated a minimum of three times.

**Figure 3:**
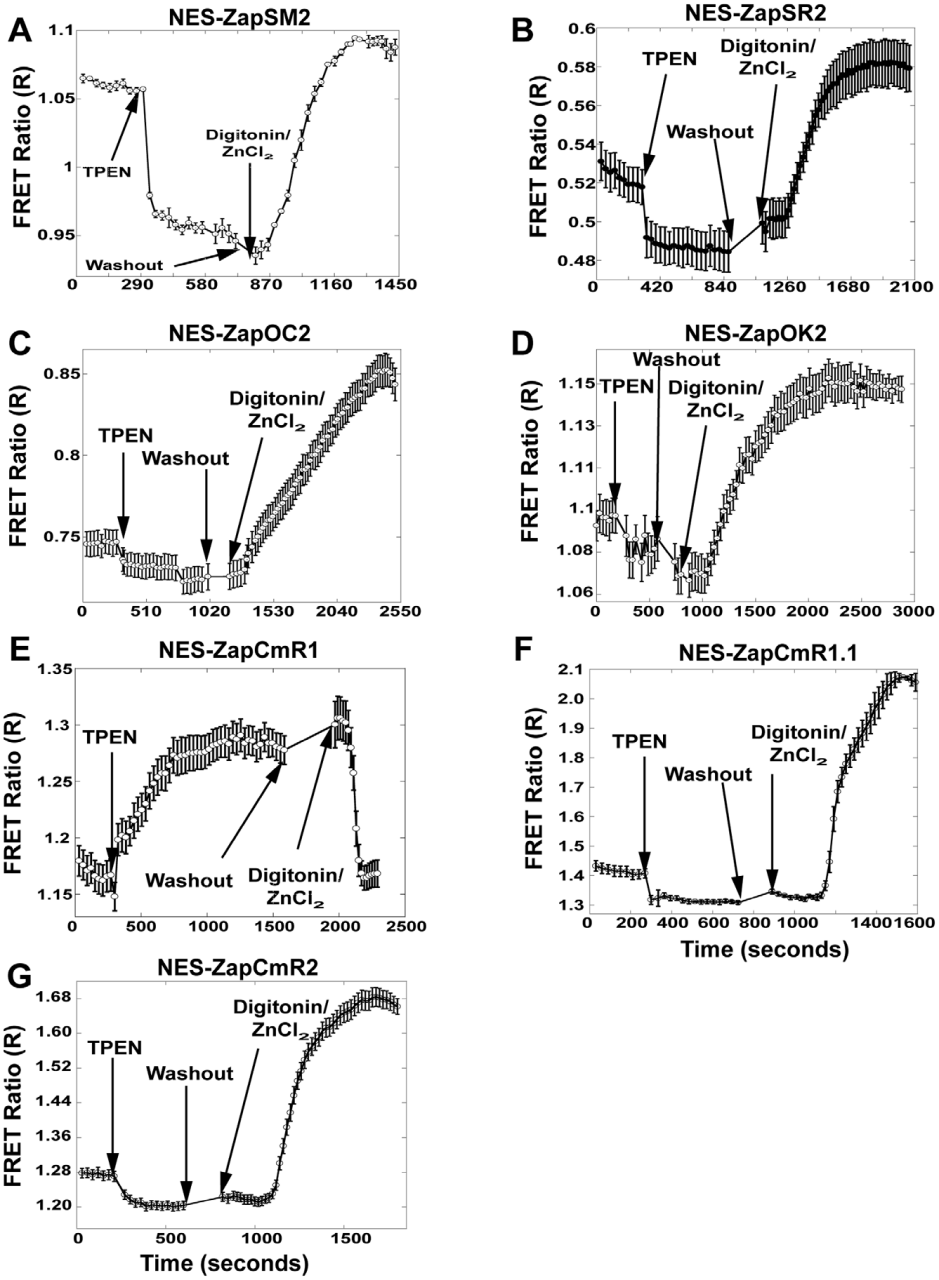
FRET Sensor calibration in the cytosol. Representative calibrations of each sensor localized to the cytosol The background corrected FRET ratio (FRET Intensity ÷ Donor Intensity) is represented as a function of time. Calibrations were performed by adding 150 µM TPEN to achieve R_TPEN_, followed by washing of residual TPEN and addition of 135 µM ZnCl_2_ with 10 µM Digitonin to permeabilize the cell membrane and obtain R_Zn_. A) NES-ZapSM2, FRET ratio goes slightly above resting; B) NES-ZapSR2 has a similar response as observed in the nucleus, Figure 2B; C) NES-ZapOC2 demonstrates a small decrease after TPEN compared to the same sensor in the nucleus; D) NES-ZapOK2 is observed with small changes in FRET ratio after TPEN and Zn^2+^/digitonin; E) NES-ZapCmR1 has an inverted response in which TPEN causes an increase in FRET ratio while Zn^2+^ with digitonin causes a decrease in the ratio; F) NES-ZapCmR1.1 and G) NES-ZapCmR2 exhibit a small decrease with TPEN and a larger increase in FRET ratio after addition of Zn^2+^ and digitonin. Representative traces are mean ± s.e.m. (n = 4 cells). Each experiment was repeated a minimum of three times.

### *In vitro* FRET Sensor Protein Purification

Plasmids containing the sensors were transformed into BL21 *E. coli*, expression was induced with 500 µM isopropyl β-D-1-thiogalactopyranoside (IPTG) (Gold Biotechnology), and sensor protein was purified by the His-tag using Ni^2+^ affinity chromatography. Purified sensor was buffer exchanged into 10 mM MOPS, 100 mM KCl pH 7.4 and absorption and emission spectra were recorded using a Tecan Safire-II fluorescence plate reader with the following parameters: ZapSM2 and ZapSR2, excitation: 380 nm, emission: 470–650 nm; ZapOC2 and ZapOK2, excitation: 525 nm, emission: 540–650 nm; ZapCmR excitation: 445 nm, emission: 470–700 nm. All measurements had an emission bandwidth of 10 nm.

**Table 2:** Comparison of sensors with different fluorescent proteins.

Sensor Name	*In vivo* Dynamic Range(R_max_/R_min_) (Mean±SEM)	Percent Saturation at Rest [(R-R_TPEN_)/(R_Zn_-R_TPEN_)x100%	R_rest_	R_max_-R_min_
NLSZapSM2	1.14±0.003	91% ±3%	0.89	0.11
NESZapSM2	1.13±0.01	67±5%	1.05	0.15
NLSZapSR2	1.18±0.004	48±3%	0.55	0.1
NESZapSR2	1.21±0.01	38±2%	0.52	0.1
NLSZapOC2	1.11±0.01	22±2%	0.88	0.12
NESZapOC2	1.13±0.01	20±2%	0.74	0.13
NLSZapOK2	1.1±0.01	32±4%	0.93	0.06
NESZapOK2	1.09±0.004	35±2%	1.09	0.08
NLSZapCmR1	1.15±0.01	92±2	1.02	0.15
NESZapCmR1	1.17±0.04	88±7	1.07	0.18
NLSZapCmR1.1	1.44±0.5	22±6	1.22	0.4
NESZapCmR1.1	1.52±0.03	17±1	1.43	0.6
NLSZapCmR2	1.38±0.02	24±1	1.38	0.4
NESZapCmR2	1.39±0.02	17±1	1.28	0.5

* Each experiment was performed in triplicate and a minimum of 3–4 cells per field of view were observed.

### Cell Culture and Microscopy

HeLa cells were grown in Dulbecco’s Modified Eagle’s Medium (DMEM) (Life Technologies) supplemented with 10% (v/v) fetal bovine serum (Atlanta Biologicals), 100 U/mL penicillin, and 100 µg/mL streptomycin. Cells were incubated at 37°C in 5% CO_2_, changing the media every 3 days. Once cells were approximately 80–90% confluent they were split and seeded onto 3.5 cm imaging dishes until they were approximately 40–50% confluent. At this point 1 µg of sensor DNA was transiently transfected using TransIT®-LT1 (Mirus) as specified by manufacturer instructions.

**Figure 4:**
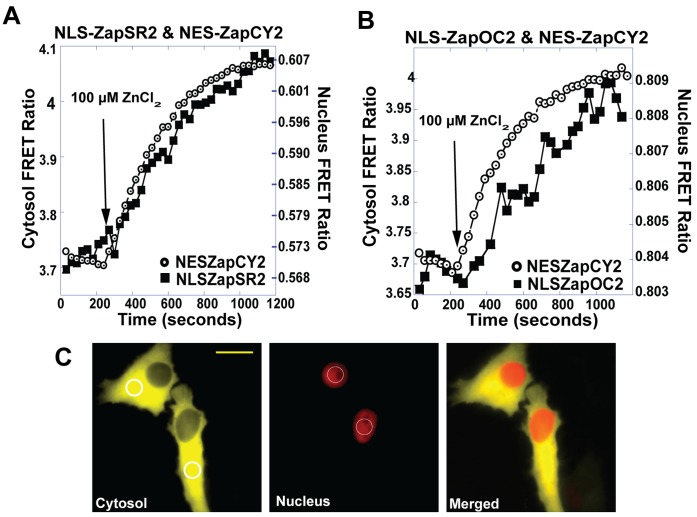
Simultaneous monitoring of cytosolic and nuclear Zn^2+^ uptake. (A) Simultaneous imaging of NLS-ZapSR2 and NES-ZapCY2 in the same cell. (B) Simultaneous imaging of NLS-ZapOC2 and NES-ZapCY2 in the same cell. In both experiments 100 µM ZnCl_2_ was added at the time indicated. The rate of increase in the FRET ratio is essentially the same in both locations, suggesting similar rates for nuclear and cytosolic uptake. C) Left panel (cytosol) is NES-ZapCY2 and circles represent ROI followed throughout experiment, middle panel represents NLS-ZapSR2, circles represent ROI (NLS-ZapOC2 not shown), and right panel represents NLS-ZapSR2 and NES-ZapCY2 merged. Images were bleedthrough corrected. Experiments were repeated at least five times with a minimum of 1–2 cells per experiment. Scale bar = 20 µm.

Forty-eight hours after transfection, cells were imaged using phosphate, calcium, and magnesium free HEPES-buffered Hanks’ balanced salt (HHBSS) media (5.36 mM KCl, 137 mM NaCl, 16.65 mM D-Glucose, and 30 mM HEPES) pH 7.4. This buffer is made in our laboratory because phosphates are known to precipitate Zn^2+^. Buffer was generated with Chelex-100-treated water (Sigma Aldrich). Cell imaging was performed on a Zeiss Axiovert 200 M microscope with a Cascade 512B CCD camera (Roper Scientific) and Xenon arc lamp (XBO75) using MetaFluor software (Universal Imaging) to operate the system. All excitation filters, dichroic mirrors, and emission filters (Chroma Technology or Semrock) are presented in **Table S2**. The following settings were used: exposure time 400 msec, 20–30 second acquisition rate, 1.3NA 40× oil immersion objective, and excitation light was attenuated with either a 10% neutral density filter for tSapphire paired with mKO or TagRFP as well as for Clover and mRuby2 and 5% neutral density filter for mOrange2 paired with mCherry or mKATE as well as for the CFP-YFP pair.

**Figure 5:**
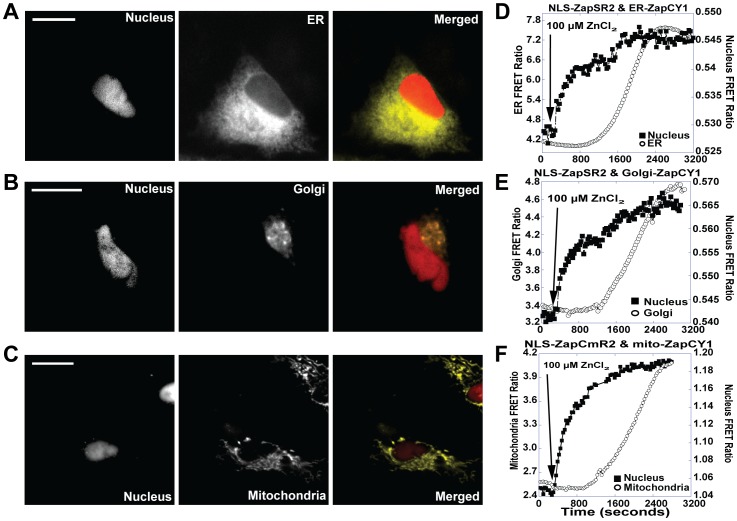
Simultaneous monitoring of Zn^2+^ uptake into the nucleus and either the endoplasmic reticulum, Golgi apparatus, or mitochondria. Representative images (FRET channel) and FRET ratio traces of Zn^2+^ uptake into the nucleus, ER, Golgi, or mitochondria. A) Image of nuclear and ER FRET sensor, left panel illustrates NLS-ZapSR2, middle panel ER-ZapCY1 and right panel is a pseudo-color merged image of NLS-ZapSR2 and ER-ZapCY1. B) Image of nuclear and ER FRET sensor, left panel illustrates NLS-ZapSR2, middle panel Golgi-ZapCY1 and right panel is a pseudo- color merged image of NLS-ZapSR2 and Golgi-ZapCY1. C) Image of nuclear and mitochondrial FRET sensor, left panel illustrates NLS-ZapCmR2, middle panel mitochondria-ZapCY1 and right panel is a pseudo- color merged image of NLS-ZapCmR2 and mitochondria-ZapCY1. D-F) FRET ratio traces of NLS-ZapSR2 or NLS-ZapCmR2 with ER-, Golgi-, and mitochondrial-ZapCY1 upon addition of 100 µM extracellular ZnCl_2_ at the time indicated. The nuclear FRET ratio rises more rapidly than organelle FRET ratio. The organelle FRET ratio begins to increase approximately 600 seconds post-Zn^2+^. Experiments were repeated at least five times with a minimum of 1–2 cells per experiment. All images were bleedthrough corrected. Scale bar = 20 µm.

### Live Cell Imaging Experiments

To characterize sensors in cells, a region of interest (ROI) was placed on an individual cell and on an untransfected cell or a region in the field of view with no cells as a measure of the background fluorescence. The FRET channel is defined as the emission intensity in the acceptor FP channel, upon excitation of the donor. The FRET ratio is defined as the background corrected intensity in the FRET channel divided by the background corrected intensity in the donor channel, i.e. (I_FRET_ - I_FRETbackground_)/(I_Donor_ - I_donorbackground_). For imaging experiments, DMEM media was removed and cells were washed twice with HHBSS buffer followed by the addition of 1 mL HHBSS. ROIs were followed for 300 seconds to establish a resting R followed by the addition of 150 µM TPEN (N,N,N′,N′-tetrakis-(2-pyridylmethyl)-ethylenediamine) to chelate Zn^2+^ and obtain the FRET ratio of the unbound sensor (R_TPEN_). Once R_TPEN_ was established, cells were washed 3-times with the HHBSS solution to remove residual TPEN followed by addition of 1 mL of fresh HHBSS. Subsequently, 135 µM ZnCl_2_ and 10 µM digitonin were added to saturate the sensor and establish the FRET ratio of the Zn^2+^ bound sensor (R_Zn_). Data were collected at 20–30 sec acquisition rate. The dynamic range of each sensor was defined as the maximum FRET ratio divided by the minimum FRET ratio (R_max_/R_min_), and the percent saturation was calculated according to [(|R-R_TPEN_|)/(|R_Zn_-R_TPEN_|)]*100%.

### Simultaneous Monitoring of Zn^2+^ Uptake into Multiple Compartments

In order to monitor Zn^2+^ in two compartments of a single cell, cells were transfected with both a cyan-yellow and green-red or red-orange FRET sensor. Zinc uptake was initiated by addition of 100 µM ZnCl_2_ extracellularly without any ionophores or membrane permeabilization agents. Previous studies in our lab have established that addition of 100 µM ZnCl_2_ extracellularly leads to a rise in cytosolic Zn^2+^ from ∼100 pM to ∼6 nM [15]. The background corrected FRET ratio was measured for each compartment as described above.

## Results

### Measurement of Spectral Bleedthrough

Given the lack of consensus on an optimal green-red or red-orange FRET pair, we decided to test 5 different alternate FRET pairs in the zinc sensor platform. **Table 1** summarizes the sensors generated using tSapphire, an ultra-violet (UV) excitable FP [18], Clover [19], mKO [20], mOrange2 [21], TagRFP [22], mCherry [23], mKATE [24], or mRuby2 [19], and **Figure S1** presents the absorption and emission spectra of purified sensor proteins. Because these FRET sensors are ratiometric, emission of the donor FP decreases while emission of the acceptor FP increases upon Zn^2+^ binding. An important consideration when evaluating whether multiple FRET sensors can be used simultaneously in cells is the extent of spectral crosstalk between individual FPs and FRET constructs. Spectral crosstalk generally refers to the presence of fluorescence signal from more than one species in a single optical channel. The extent of crosstalk is given by the percent bleedthrough (or contamination) of a given fluorescent species in an optical channel. Because FRET ratios are calculated using the FRET optical channel and the donor optical channel, we felt it was important to determine bleedthrough in both of these channels. For the donor channel, HeLa cells were transfected with cDNA encoding an individual FP and the fluorescence intensity was measured in all channels. **Table S3 and Figures S2 and S3** present the percent bleedthrough in each of the donor channels (CFP, tSapphire, mOrange2, and Clover), which was less than 5% in all cases. For the FRET channel, constructs in **Table 1** were transfected into cells and the intensity was measured in each of the FRET channels (**Figure S4 and Table S4**). When pairing CFP-YFP with a green-red sensor, there was substantial bleedthrough of Clover-mRuby2, tSapphire-TagRFP and tSapphire-mKO into the CFP-YFP FRET channel (50%, 96%, and 80%, respectively), and<10% of CFP-YFP into the green-red channels. This indicates that if one of these green-red sensors is to be used alongside a CFY-YFP sensor, the sensors need to be in non-overlapping spatial locations. Spectral crosstalk was minimized by pairing CFP-YFP with an orange-red sensor (<3% for all combinations).

### Generation of Nuclear and Cytosol Localized Constructs

There are no published sensors for quantitatively measuring Zn^2+^ in the nucleus. Yet nuclear Zn^2+^ is an important component of cellular Zn^2+^ homeostasis. First, a large number of nuclear proteins, including polymerases, transcription factors, and DNA remodeling factors such as histone deacetylases require Zn^2+^ for proper function [25], [26]. Second, there is evidence that the primary Zn^2+^ storage protein, metallothionein, shuttles into the nucleus during the G_1_ to S phase transition, suggesting dynamic regulation of nuclear Zn^2+^ during cell division [27]. Third, studies have reported the generation of transient nuclear Zn^2+^ signals [28]. Therefore, we thought it would be valuable to generate both nuclear- localized and cytosplasmic Zn^2+^ sensors of the non-CFP/YFP variety. **Figure 1a** shows a schematic of the sensor construct illustrating the localization signals. **Figure 1b** shows a representative FRET sensor localized to either the nucleus or the cytosol. All sensors exhibited a similar localization pattern.

### Characterization of Sensors in HeLa Cells

There are many examples of genetically encoded biosensors exhibiting diminished responses in cells compared to in vitro [15], [16] therefore we set out to screen all sensors in mammalian cells to verify functionality. All seven sensors described in **Table 1** were transiently transfected into HeLa cells, expressed in either the nucleus or cytosol, and subjected to an in situ calibration to determine R_resting_, R_TPEN_, and R_Zn_. **Figure 2** shows that all nuclear-localized sensors responded to manipulation of cellular Zn^2+^, with the majority of sensors exhibiting an increase in the FRET ratio for R_Zn_ and a decrease for R_TPEN_. ZapCmR1 was the only sensor that displayed an inverted response (R_TPEN_>R_Zn_). It is not uncommon for sensors to exhibit inverted FRET responses when the relative orientation of the FPs is altered [17], particularly when the linkers are different as they are in the Clover-mRuby2 construct. Incorporation of mutations in the ZBD reverted the response to that of the other sensors, and decreased the affinity for Zn^2+^ as observed by comparison of R_TPEN_ and R_Zn_ with other sensors. **Figure 3** shows that all sensors localized to the cytosol responded to manipulation of cellular Zn^2+^.

**Table 2** presents the dynamic range for each sensor, which varies from 1.1 to 1.2-fold for most of sensors with the exception of ZapCmR1.1 and ZapCmR2 which exhibit a 1.4–1.5 fold change. Two additional important parameters are the resting FRET ratio and the R_max_ – R_min_ which help to define the signal-to-noise. For example if the dynamic range is 1.1 and the resting ratio is 0.5, this means the FRET ratio only changes from 0.5 to 0.55, i.e. R_max_ – R_min_ is 0.05; whereas if the resting ratio is 1.0, the same dynamic range would yield a FRET ratio change from 1 to 1.1 and hence an R_max_ – R_min_ of 0.1 and overall greater sensitivity.

The percent saturation is a measure of how much Zn^2+^ is bound to a sensor under resting conditions and provides a relative measure of Zn^2+^ levels in different locations. **Table 2** shows the resting percent saturation of each sensor in the nucleus and cytosol. Interestingly six sensors reveal a higher saturation percentage in the nucleus than in the cytosol, suggesting that perhaps nuclear Zn^2+^ is buffered at a higher concentration than the cytosol.

### Zinc Uptake into the Cytosol and Nucleus

Extracellular Zn^2+^ levels are typically in the 1–10 µM range [29], [30], [31], but a number of cells contain high levels of Zn^2+^ in vesicles and secrete Zn^2+^ in response to stimulation [32], [33], [34], [35]. Therefore, there are physiological situations in which extracellular Zn^2+^ is transiently elevated. We have demonstrated that elevation of extracellular Zn^2+^ results in uptake of Zn^2+^ into the cytosol [15], but it is unclear whether this translates into an increase in nuclear Zn^2+^. Therefore we set out to monitor Zn^2+^ uptake in both the cytosol and nucleus with the new sensors. **Figure S5** depicts representative traces of each sensor in the cytosol upon elevation of extracellular Zn^2+^, confirming with the new sensors are sensitive enough for monitoring Zn^2+^ uptake. **Figure S6** demonstrates that all nuclear sensors exhibit an increase in the FRET ratio, indicating that nuclear Zn^2+^ also rises under this experimental paradigm. Because ZapCmR1 was close to saturated under resting conditions, it was not used for uptake studies.

While the Clover-mRuby2 sensors clearly represent superior green-red sensors, we wanted to test the limits of responsiveness of the low dynamic range sensors. Therefore, we cotransfected these sensors with a cytosolic CFP-YFP sensor to simultaneously monitor Zn^2+^ uptake into the nucleus and cytosol. **Figure 4** reveals that two sensors (NLS-ZapSR2 and -ZapOC2) were sensitive enough to detect changes in nuclear Zn^2+^ when coupled with cytosolic ZapCY2. Moreover, under this experimental paradigm, the cytosol and nucleus accumulated Zn^2+^ with comparable rates, indicating that in defining the rate of Zn^2+^ uptake from the extracellular environment, localizing sensors to the nucleus could serve as a proxy for monitoring the rate of change of cytosolic Zn^2+^. NLS-ZapSR2 exhibited the largest FRET ratio change making it the preferable choice of low sensitivity sensors.

### Simultaneous Monitoring of Nuclear and Organelle Zn^2+^ Uptake

Previous studies in our lab have demonstrated that intracellular organelles such the ER, Golgi, and mitochondria can accumulate Zn^2+^ when cytosolic Zn^2+^ levels become elevated, demonstrating these compartments play an important role in cytosolic clearance and maintenance of homeostasis [15], [36]. However for these prior experiments accumulation of Zn^2+^ in the cytosol and intracellular organelles was measured in separate experiments making it impossible to directly compare the relative rate of uptake in individual cells. To observe Zn^2+^ uptake into the nucleus we used either NLS-ZapSR2 or NLS-ZapCmR2 and ZapCY1 was targeted to individual organelles. **Figure 5a–c** depicts representative images showing sensor localization. As observed in **Figure 5d–f**, addition of 100 µM ZnCl_2_ to the extracellular milieu, gave rise to an immediate increase in nuclear Zn^2+^, whereas organelle Zn^2+^ (ER, Golgi, and mitochondria, **Figure 5d, 5e, and 5f**) increases approximately 600–700 seconds later.

### Conclusions

Here we report the first alternatively colored Zn^2+^ sensors constructed from green, orange, and red FPs that can be used simultaneously with a CFP/YFP sensor. Given evidence that changes in cellular Zn^2+^ have been linked with changes in other ions such as Ca^2+^ [15], [37] and signaling pathways such as the MAPK pathway [38] and apoptotic cascades [39], [40], a broad palette of Zn^2+^ sensors that permits simultaneous monitoring of multiple events would be useful tools to provide mechanistic insight into these connections.

Sensors were targeted to both the cytosol and nucleus and intriguingly, 6 of the 7 sensors registered a higher fractional saturation in the nucleus compared to the cytosol, suggesting that nuclear Zn^2+^ may be buffered at a higher concentration than cytosolic Zn^2+^. Although there are currently no estimates of nuclear Zn^2+^ levels, given the large number of transcription factors that bind Zn^2+^ [41], it seems reasonable to speculate the nuclear buffering system may differ from that in the cytosol.

The development of multi-color FRET sensors for Zn^2+^ allowed us to monitor Zn^2+^ simultaneously in the nucleus and other organelles, such as the ER, Golgi, or mitochondria. For these experiments we measured Zn^2+^ uptake or sequestration following acute elevation of extracellular Zn^2+^. Extracellular Zn^2+^ levels are typically in the range of 1 to 10 µM. However there are a number of cell types (hippocampal neurons, prostate cells, and pancreatic cells) that are known to secrete Zn^2+^. Although the amount of Zn^2+^ these cells secrete has not been rigorously quantified, estimates range from 1 µM to 2 mM [32], [33], [34], [35]. Therefore we believe the acute elevation of extracellular Zn^2+^ represents a physiologically relevant stimulus, similar to Zn^2+^ secretion. Here we demonstrate that elevation of extracellular Zn^2+^ leads to immediate uptake of Zn^2+^ into the nucleus and much slower sequestration of Zn^2+^ into organelles after a lag time of ∼ 600 sec. The reason for organelle sequestration is unclear but it may represent a detoxification mechanism, ensuring that cytosolic levels of Zn^2+^ don’t get too high. The mechanism of sequestration and the reason for the delay are unclear. There are 24 mammalian zinc transporters with varied subcellular distribution and it is possible that specific transporters are activated for Zn^2+^ transport into organelles [42]. We believe these new tools (i.e. sensors targeted to multiple locations to simultaneously monitor Zn^2+^ flux) will allow us to dissect these fundamental questions about Zn^2+^ homeostasis.

In contrast to the robust signal that is observed using CFP and YFP as a FRET pair, alternate color FRET pairs often suffer from greatly reduced dynamic ranges. Additionally, because of the lower quantum yields of most red FPs compared to appropriate donor FPs and the relatively poor sensitized emission, FRET sensors with a red FP as the acceptor typically yield a resting FRET ratio less than 1 and a reduced ΔR, thus limiting their sensitivity. Not surprisingly the majority of sensors reported here exhibit modest FRET ratio changes. Of the 5 different FRET pairs explored, Clover-mRuby2 based sensors yielded the greatest sensitivity with a dynamic range of 1.4–1.5 and ΔR of 0.4–0.6. The other FRET sensors exhibited a lower ΔR making them less robust for monitoring cellular Zn^2+^ fluxes, although many were still sensitive enough to permit measurement of subtle changes in cellular Zn^2+^ levels. Still, ZapCmR1.1 and ZapCmR2 are clearly superior and even exhibit a slightly better dynamic range than their CFP-YFP counterpart, ZapCY2 [15].

In conclusion, we have established new genetically encoded Zn^2+^ sensors employing FRET pairs that are complementary to the traditional CFP-YFP pair that can help define Zn^2+^ dynamics in different compartments simultaneously. A limitation of some of these alternately colored biosensors is that their dynamic range is reduced compared to their CFP-YFP counterparts (1.1–1.2 versus 1.3–1.4). However, we did identify superior sensors based on the new FRET pair Clover and mRuby2, which have higher dynamic ranges than their CFP-YFP counterparts. These green-red sensors should also be useful for monitoring Zn^2+^ levels alongside other signaling agents such as Ca^2+^ and therefore have the potential to be instrumental in dissecting crosstalk between these two ions.

## Supporting Information

Figure S1 **Absorption and Emission Spectra of Purified Sensor Protein.** Scans of A) ZapSM2, B) ZapSR2, C) ZapOC2, D) ZapOK2, and E) ZapCmR2. Plots represent absorption spectra of each FRET sensor (red traces); emission spectra in the presence of Zn^2+^ (∼150 µM - green traces) and in the absence of Zn^2+^ (1 µM EGTA - blue traces). For excitation and emission parameters refer to materials and methods section of the text. Given the published molar extinction coefficient and quantum yield ZapCmR2 is proteolyzed resulting in a small mRuby2 FRET emission peak.
(PDF)

Figure S2 **Bleed-through of fluorescent proteins.** Representative images for bleedthrough measurements. Cells were transfected with the FP listed on the left hand side and the fluorescence intensity in channels A through F were measured. Ex = Excitation and Em = Emission in nanometers.
(PDF)

Figure S3 **Bleed-through of fluorescent proteins.** Representative images for bleedthrough measurements. Cells were transfected with the FP listed on the left hand side and the fluorescence intensity in channels A through F were measured. Ex = Excitation and Em = Emission in nanometers. Scale bar = 20 µm.
(PDF)

Figure S4 **Cross-talk of FRET sensors.** Representative images for bleedthrough measurements. Cells were transfected with the FRET sensor listed on the left hand side and the fluorescence intensity in channels A through D were measured. Ex = Excitation and Em = Emission in nanometers. Scale bar = 20 µm.
(PDF)

Figure S5 **Zn^2+^ uptake into nucleus.** Sensors were localized to nucleus to monitor uptake of extracellular Zn^2+^. A) NLS-ZapSM2, B) NLS-ZapSR2, C) NLS-ZapOC2, D) NLS-ZapCmR1.1, E) NLS-ZapCmR2, Plots represent FRET Ratio traces of each compartment. Regions were imaged for approximately 300 seconds followed by the addition of 100 µM extracellular ZnCl_2_ at the time indicated. Nuclear FRET ratios rose immediately after the addition of Zn^2+^. The background corrected FRET ratio (FRET Intensity ÷ Donor Intensity) is represented as a function of time. Each experiment was repeated a minimum of three times with a minimum of 3–4 cells per field of view.
(PDF)

Figure S6 **Zn^2+^ uptake into cytosol.** Sensors were localized to cytosol to monitor uptake of extracellular Zn^2+^. A) NES-ZapSM2, B) NES-ZapSR2, C) NES-ZapOC2, D) ZapOK2, E) NES-ZapCmR1.1, and F) NES-ZapCmR2. Plots represent FRET Ratio traces of each compartment. Regions were imaged for approximately 300 seconds followed by the addition of 100 µM extracellular ZnCl_2_ at the time indicated. Cytosolic FRET ratios rose immediately after the addition of Zn^2+^. The background corrected FRET ratio (FRET Intensity ÷ Donor Intensity) is represented as a function of time. Each experiment was repeated a minimum of three times with a minimum of 3–4 cells per field of view.
(PDF)

Table S1 **Amino acid sequence of Zap Zinc Binding Domains (ZBD).** Zap1 disassociation constant (K_d_) = 2.53 pM; Zap2 (K_d_) = 811 pM; Zap1.1 (K_d_) = undetermined.
(DOCX)

Table S2 **Filter sets and dichroic mirrors used for cellular imaging.** For FRET experiments, the donor excitation filter and dichroic mirror are used along with the emission filter of the acceptor FP.
(DOCX)

Table S3 **Percent Bleedthrough of Fluorescent Proteins.** Each experiment was performed in triplicate and a minimum of 6-cells per field of view were observed. Values reported represent the mean ± SEM. Excitation filters, Dichroic mirrors, and Emission filters for each channel are given in Table S1. ^1^ Percent intensity was calculated as follows: Intensity in the designated channel divided by Intensity in the channel of the transfected FP. Cross-talk between the donor channels is highlighted in bold.
(DOCX)

Table S4 **Percent Bleedthrough of sensor into FRET channels.** Each experiment was performed in triplicate and a minimum of 4-cells per field of view were observed. Values reported represent the mean ± SEM. Cells were transfected with FRET sensor listed in the left column. The sensors were excited with their respective excitation filters and the emission intensity in each of the channels on the right hand side was measured. Excitation filters, Dichroic mirrors, and Emission filters for each channel are given in Table S3. Percent intensity was calculated as follows: Intensity of the designated channel divided by Intensity in the channel of the transfected FRET sensor.
(DOCX)
