# Supplementary figures and images for: New Alternately Colored FRET Sensors for Simultaneous Monitoring of Zn2+ in Multiple Cellular Locations
Source: PLoS One. 2012 Nov 16;7(11):e49371. doi: 10.1371/journal.pone.0049371 (PMC3500285; doi:10.1371/journal.pone.0049371)

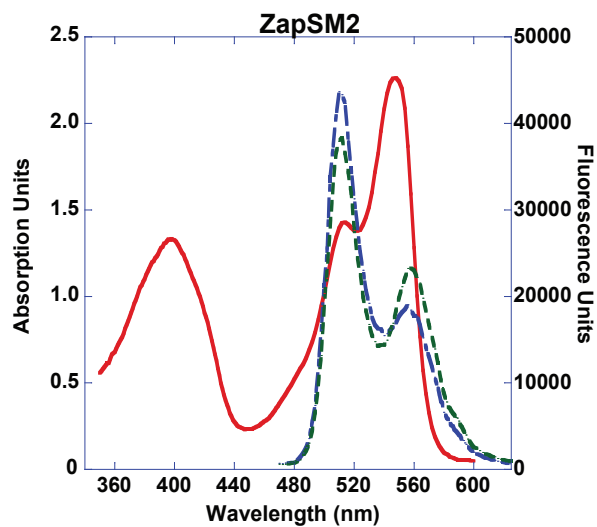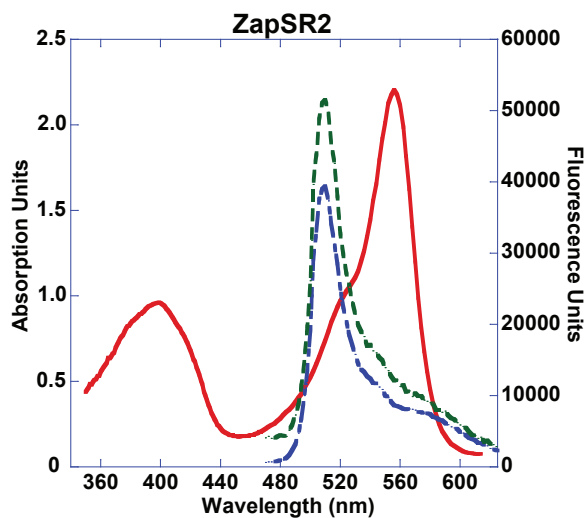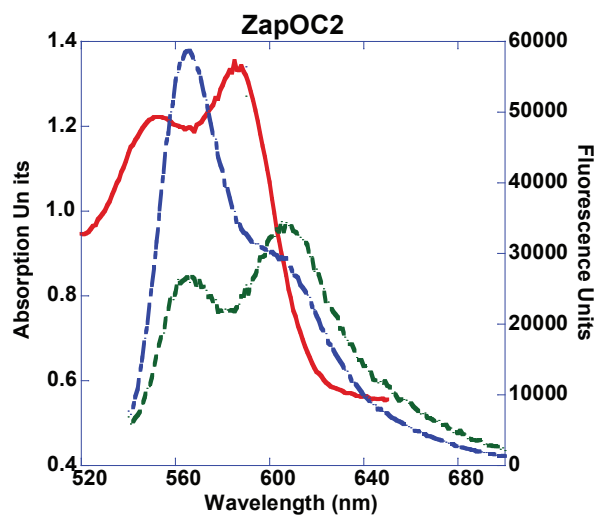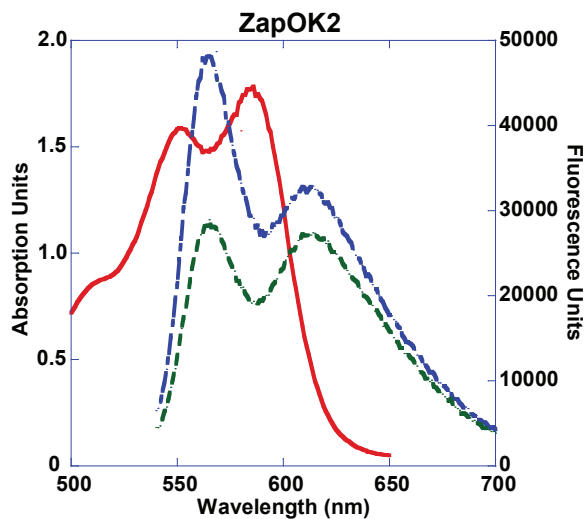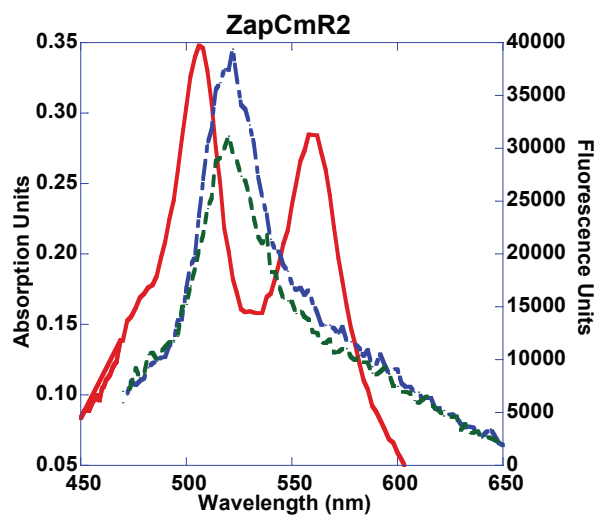

— Absorption Spectra  
- - Emission Spectra (No-Zinc)  
- - Emission Spectra (Zinc)

Supplement: Figure S1 — Absorption and Emission Spectra of Purified Sensor Protein. Scans of A) ZapSM2, B) ZapSR2, C) ZapOC2, D) ZapOK2, and E) ZapCmR2. Plots represent absorption spectra of each FRET sensor (red traces); emission spectra in the presence of Zn2+ (∼150 µM - green traces) and in the absence of Zn2+ (1 µM EGTA - blue traces). For excitation and emission parameters refer to materials and methods section of the text. Given the published molar extinction coefficient and quantum yield ZapCmR2 is proteolyzed resulting in a small mRuby2 FRET emission peak. (PDF) [file pone.0049371.s001.pdf]

# Fluorescent Protein in Cells

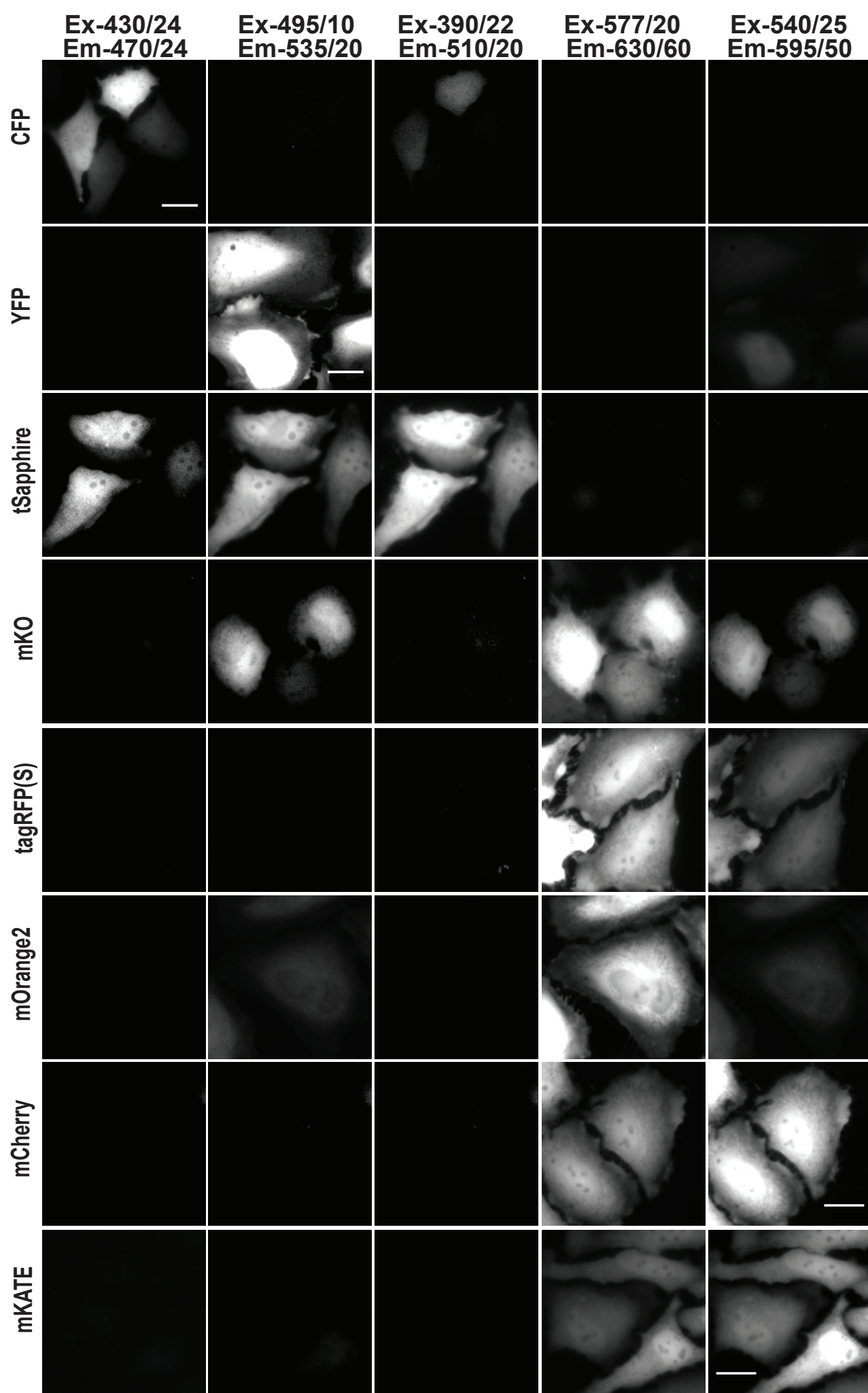

Supplement: Figure S2 — Bleed-through of fluorescent proteins. Representative images for bleedthrough measurements. Cells were transfected with the FP listed on the left hand side and the fluorescence intensity in channels A through F were measured. Ex = Excitation and Em = Emission in nanometers. (PDF) [file pone.0049371.s002.pdf]

Fluorescent Protein in Cells

Clover

Ex-480/20  
Em-510/20

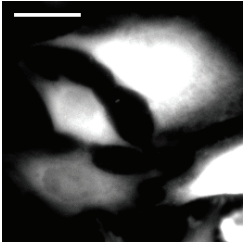

Ex-430/24  
Em-470/24

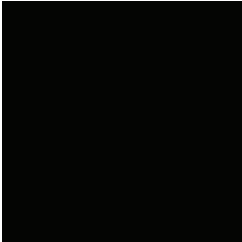

Ex-495/10  
Em-535/20

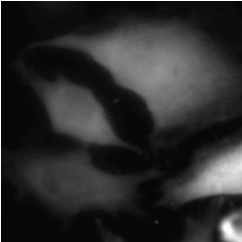

Ex-390/22  
Em-510/20

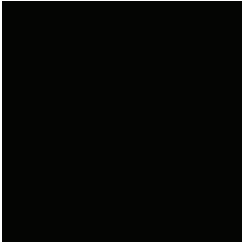

Ex-577/20  
Em-630/60

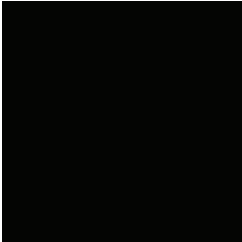

Ex-540/25  
Em-595/50

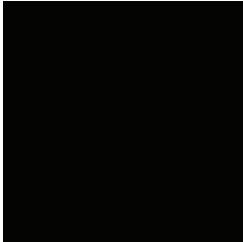

mRuby2

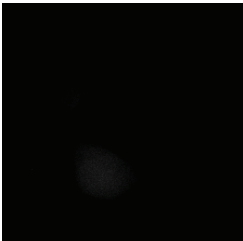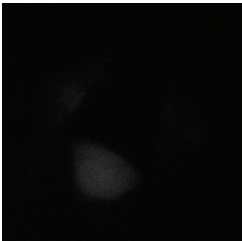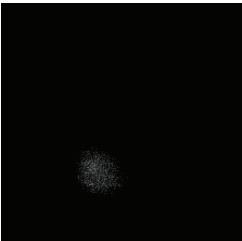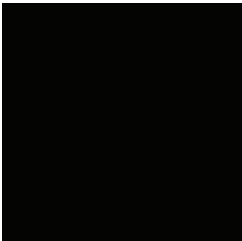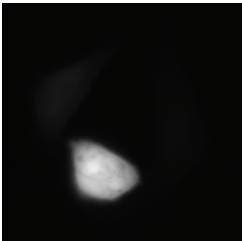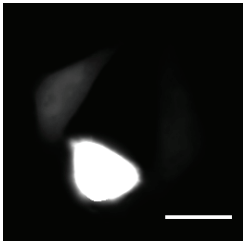

Supplement: Figure S3 — Bleed-through of fluorescent proteins. Representative images for bleedthrough measurements. Cells were transfected with the FP listed on the left hand side and the fluorescence intensity in channels A through F were measured. Ex = Excitation and Em = Emission in nanometers. Scale bar = 20 µm. (PDF) [file pone.0049371.s003.pdf]

# FRET Sensor in Cells

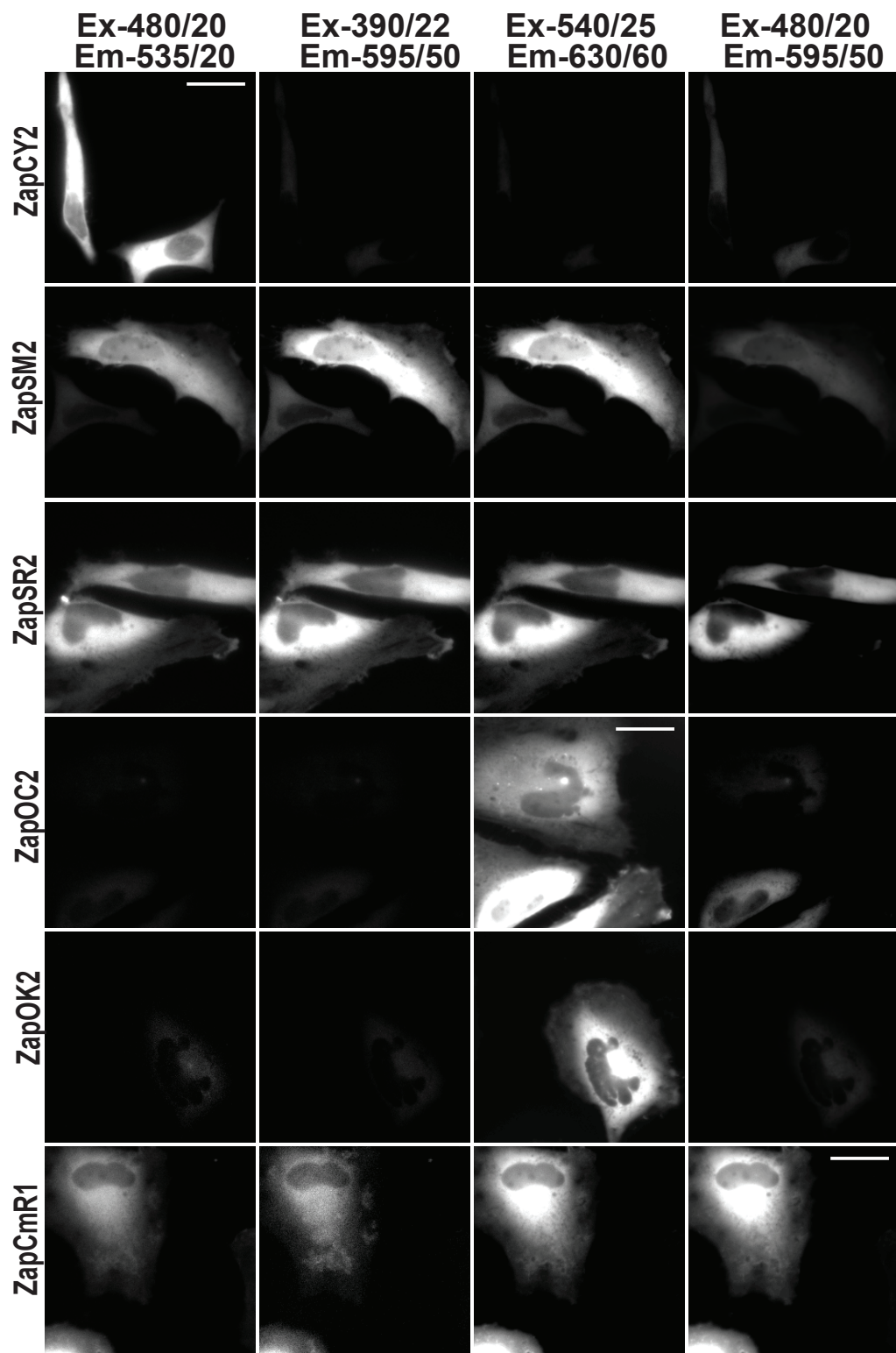

Supplement: Figure S4 — Cross-talk of FRET sensors. Representative images for bleedthrough measurements. Cells were transfected with the FRET sensor listed on the left hand side and the fluorescence intensity in channels A through D were measured. Ex = Excitation and Em = Emission in nanometers. Scale bar = 20 µm. (PDF) [file pone.0049371.s004.pdf]

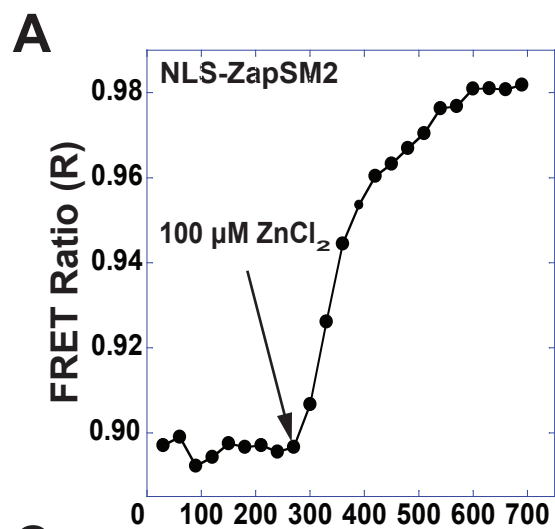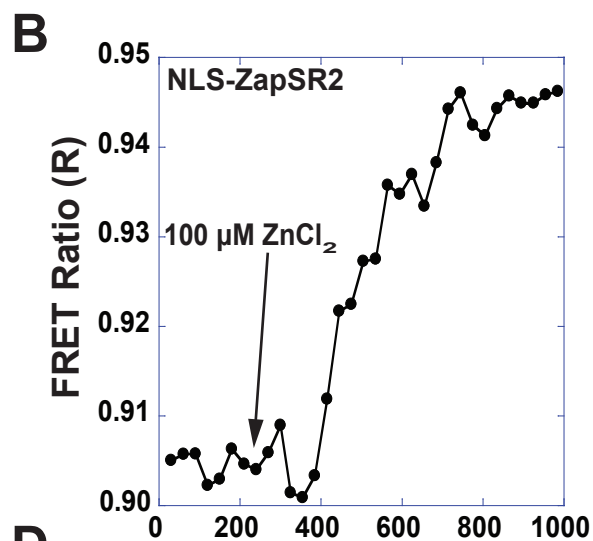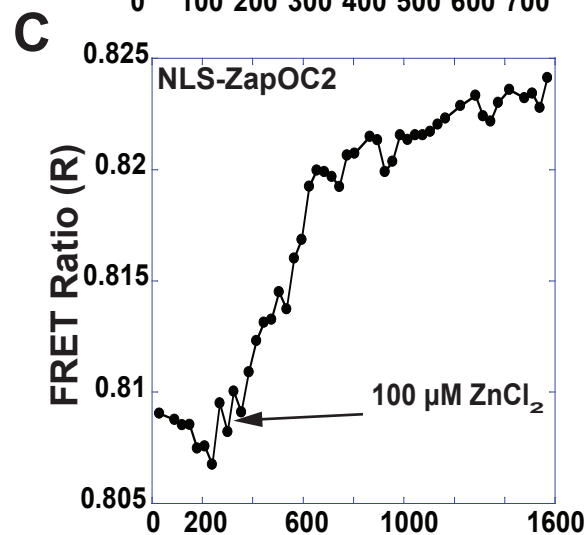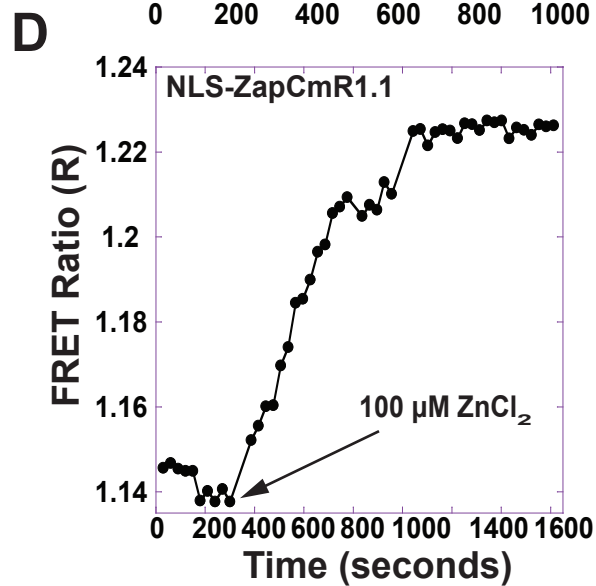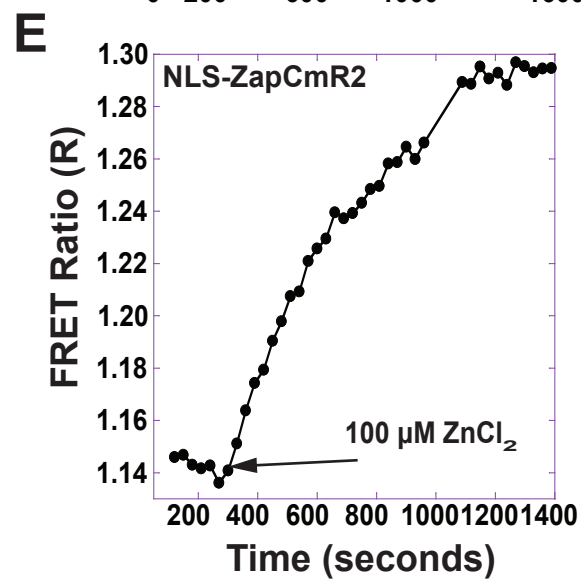

Supplement: Figure S5 — Zn2+ uptake into nucleus. Sensors were localized to nucleus to monitor uptake of extracellular Zn2+. A) NLS-ZapSM2, B) NLS-ZapSR2, C) NLS-ZapOC2, D) NLS-ZapCmR1.1, E) NLS-ZapCmR2, Plots represent FRET Ratio traces of each compartment. Regions were imaged for approximately 300 seconds followed by the addition of 100 µM extracellular ZnCl2 at the time indicated. Nuclear FRET ratios rose immediately after the addition of Zn2+. The background corrected FRET ratio (FRET Intensity ÷ Donor Intensity) is represented as a function of time. Each experiment was repeated a minimum of three times with a minimum of 3–4 cells per field of view. (PDF) [file pone.0049371.s005.pdf]

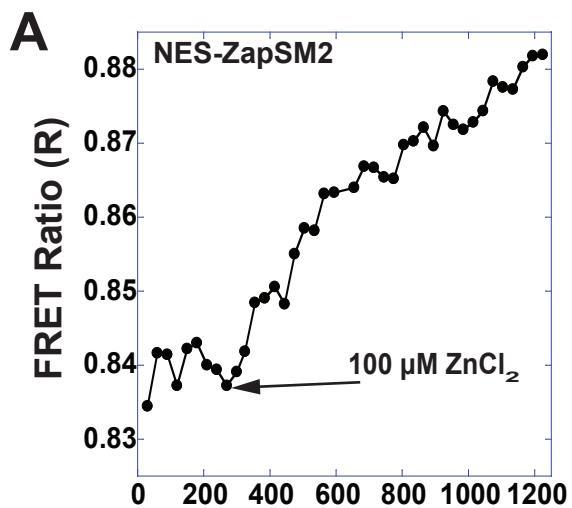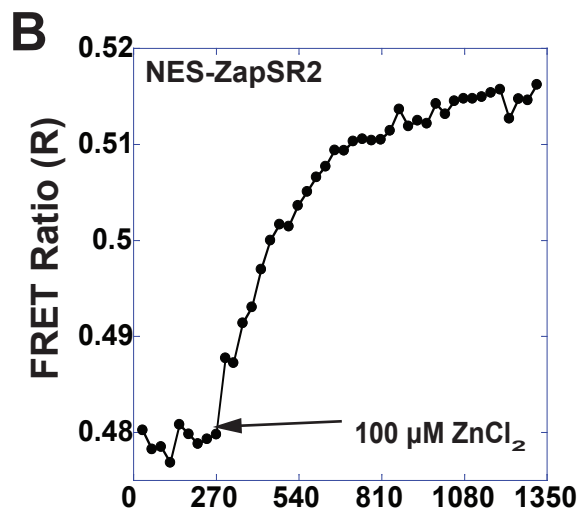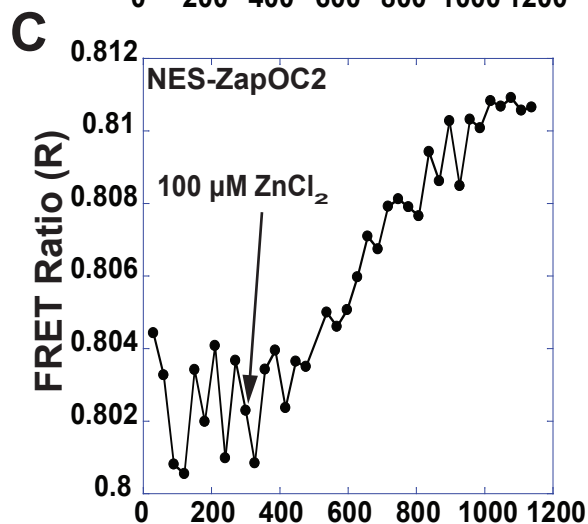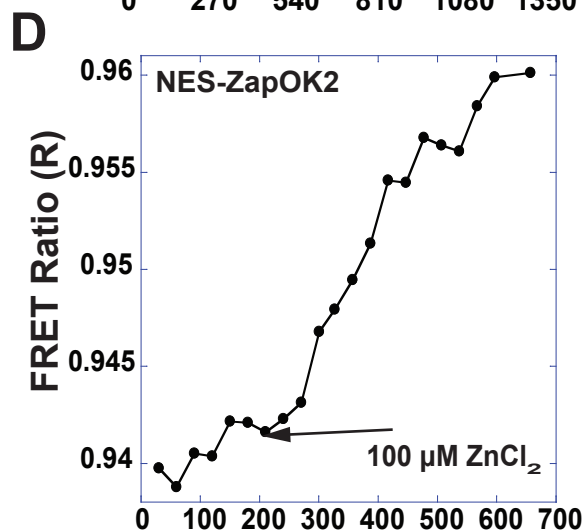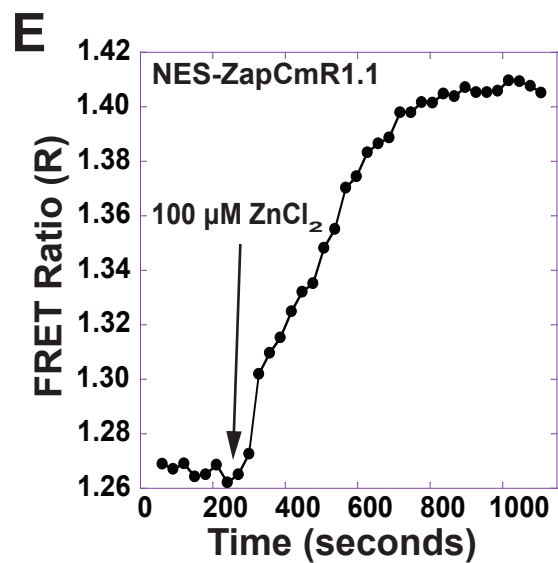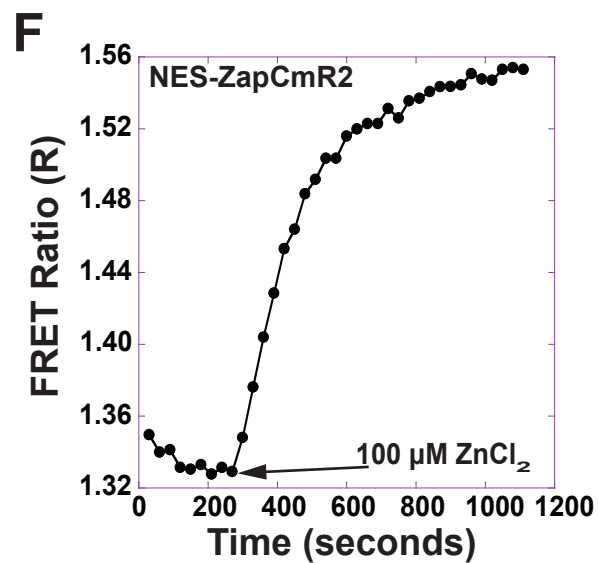

Supplement: Figure S6 — Zn2+ uptake into cytosol. Sensors were localized to cytosol to monitor uptake of extracellular Zn2+. A) NES-ZapSM2, B) NES-ZapSR2, C) NES-ZapOC2, D) ZapOK2, E) NES-ZapCmR1.1, and F) NES-ZapCmR2. Plots represent FRET Ratio traces of each compartment. Regions were imaged for approximately 300 seconds followed by the addition of 100 µM extracellular ZnCl2 at the time indicated. Cytosolic FRET ratios rose immediately after the addition of Zn2+. The background corrected FRET ratio (FRET Intensity ÷ Donor Intensity) is represented as a function of time. Each experiment was repeated a minimum of three times with a minimum of 3–4 cells per field of view. (PDF) [file pone.0049371.s006.pdf]
