# Supplementary material for: New Alternately Colored FRET Sensors for Simultaneous Monitoring of Zn2+ in Multiple Cellular Locations
Source: PLoS One. 2012 Nov 16;7(11):e49371. doi: 10.1371/journal.pone.0049371 (PMC3500285; doi:10.1371/journal.pone.0049371)
Supplement: Table S1 — Amino acid sequence of Zap Zinc Binding Domains (ZBD). Zap1 disassociation constant (Kd) = 2.53 pM; Zap2 (Kd) = 811 pM; Zap1.1 (Kd) = undetermined. (DOCX) [file pone.0049371.s007.docx]

Table S1. Amino acid sequence of Zap Zinc Binding Domains (ZBD)

| ZBD | Amino Acid Sequence |
| --- | --- |
| Zap1 | KNNDLK**C**KWKE**C**PESCSSLFDLQR**H**LLKD**H**VSQDFKHPMEPLA**C**NWED**C**DFLGDDTCSIVN**H**INCQ**H**GI |
| Zap1.1 | KNNDLK**H**KWKE**C**PESCSSLFDLQR**H**LLKD**H**VSQDFKHPMEPLA**C**NWED**C**DFLGDDTCSIVN**H**INCQ**H**GI |
| Zap2 | KNNDLK**H**KWKE**C**PESCSSLFDLQR**H**LLKD**H**VSQDFKHPMEPLA**H**NWED**C**DFLGDDTCSIVN**H**INCQ**H**GI |
