# Supplementary material for: New Alternately Colored FRET Sensors for Simultaneous Monitoring of Zn2+ in Multiple Cellular Locations
Source: PLoS One. 2012 Nov 16;7(11):e49371. doi: 10.1371/journal.pone.0049371 (PMC3500285; doi:10.1371/journal.pone.0049371)
Supplement: Table S2 — Filter sets and dichroic mirrors used for cellular imaging. For FRET experiments, the donor excitation filter and dichroic mirror are used along with the emission filter of the acceptor FP. (DOCX) [file pone.0049371.s008.docx]

Table S2. Filter sets and dichroic mirrors used for cellular imaging

| FP | Excitation filter (center/bandwidth, in nm) | Dichroic (nm) | Emission filter (center/bandwidth, in nm) |
| --- | --- | --- | --- |
| CFP | 430/24 | 455 | 470/24 |
| YFP | 495/10 | 515 | 535/20 |
| tSapphire | 390/22 | 495 | 510/20 |
| mKO | 540/25 | 565 | 595/50 |
| TagRFP | 540/25 | 565 | 595/50 |
| mOrange2 | 540/25 | 565 | 595/50 |
| mCherry | 577/20 | 595 | 630/60 |
| mKATE | 577/20 | 595 | 630/60 |
| Clover | 480/20 | 495 | 510/20 |
| mRuby2 | 540/25 | 565 | 595/50 |
