# Supplementary material for: New Alternately Colored FRET Sensors for Simultaneous Monitoring of Zn2+ in Multiple Cellular Locations
Source: PLoS One. 2012 Nov 16;7(11):e49371. doi: 10.1371/journal.pone.0049371 (PMC3500285; doi:10.1371/journal.pone.0049371)
Supplement: Table S3 — Percent Bleedthrough of Fluorescent Proteins. Each experiment was performed in triplicate and a minimum of 6-cells per field of view were observed. Values reported represent the mean ± SEM. Excitation filters, Dichroic mirrors, and Emission filters for each channel are given in Table S1. 1 Percent intensity was calculated as follows: Intensity in the designated channel divided by Intensity in the channel of the transfected FP. Cross-talk between the donor channels is highlighted in bold. (DOCX) [file pone.0049371.s009.docx]

Table S3. Percent Bleedthrough of Fluorescent Proteins

| Transfected FP | Percent intensity in each channel^1^ | | | | | |
| --- | --- | --- | --- | --- | --- | --- |
|  | Clover channel | CFP channel | YFP channel | tSapphire channel | mKO/mOrange2/TagRFP/mRuby2  channel | mCherry/mKATE  channel |
| CFP | 15 ± 0.06 | 100 | 0.06 ± 0.01 | **3 ± 0.03** | **0.09 ± 0.01** | 0.06 ± 0.01 |
| YFP | 41 ± 0.6 | 0.02 ± 0.003 | 100 | 0.06 ± 0.001 | 5 ± 0.3 | 0.01 ± 0.01 |
| tSapphire | 16 ± 0.06 | **4 ± 0.06** | 27 ± 0.2 | 100 | 0.01 ± 0.03 | 0.3 ± 0.03 |
| mKO | 2 ± 0.4 | 0.4 ± 0.05 | 4 ± 0.07 |  | 100 | 2 ± 0.08 |
| mOrange2 | 0.2 ± 0.01 | **0.3 ± 0.01** | 4 ± 0.05 | 0.07 ± 0.01 | 100 | 4 ± 0.02 |
| TagRFP | 0.09 ± 0.01 | 0.6 ± 0.08 | 0.3 ± 0.03 | 0.1 ± 0.01 | 100 | 22 ± 0.04 |
| mCherry | 0.16 ± 0.02 | 0.4 ± 0.02 | 0.06 ± 0.01 | 0.08 ± 0.01 | 57 ± 0.1 | 100 |
| mKATE | 0.35 ± 0.03 | 0.5 ± 0.02 | 2 ± 0.01 | 0.2 ± 0.01 | 43 ± 0.3 | 100 |
| Clover | 100 | **0.3 ± 0.03** | 84 ± 5.3 | 0.2 ± 0.01 | 0.3 ± 0.001 | 0.06 ± 0.004 |
| mRuby2 | 3 ± 0.3 | 8 ± 1 | 2 ± 0.2 | 0.3 ± 0.07 | 100 | 44 ± 0.1 |
