# Supplementary material for: New Alternately Colored FRET Sensors for Simultaneous Monitoring of Zn2+ in Multiple Cellular Locations
Source: PLoS One. 2012 Nov 16;7(11):e49371. doi: 10.1371/journal.pone.0049371 (PMC3500285; doi:10.1371/journal.pone.0049371)
Supplement: Table S4 — Percent Bleedthrough of sensor into FRET channels. Each experiment was performed in triplicate and a minimum of 4-cells per field of view were observed. Values reported represent the mean ± SEM. Cells were transfected with FRET sensor listed in the left column. The sensors were excited with their respective excitation filters and the emission intensity in each of the channels on the right hand side was measured. Excitation filters, Dichroic mirrors, and Emission filters for each channel are given in Table S3. Percent intensity was calculated as follows: Intensity of the designated channel divided by Intensity in the channel of the transfected FRET sensor. (DOCX) [file pone.0049371.s010.docx]

Table S4. Percent Bleedthrough of sensor into FRET channels

| Transfected Sensor | Percent intensity in emission channels below upon direct excitation of the transfected FRET Sensor^1^ | | | |
| --- | --- | --- | --- | --- |
|  | Cyan-yellow | tSapphire-mKO  tSapphire-TagRFP | mOrange-mCherry  mOrange-mKATE | Clover-mRuby2 |
| ZapCY2 | 100 | 4.7 ± 0.02 | 2.17 ± 0.01 | 9.4 ± 0.1 |
| ZapSM2 | 80 ± 1 | 100 | ≥ 100 | 37 ± 0.3 |
| ZapSR2 | 96 ± 2 | 100 | ≥ 100 | 31 ± 0.2 |
| ZapOC2 | 2 ± 0.2 | 3.5 ± 0.1 | 100 | 17.5 ± 0.4 |
| ZapOK2 | 2.3 ± 0.1 | 2.8 ± 0.1 | 100 | 17 ± 0.3 |
| ZapCmR1 | 50 ± 1 | 11 ± 0.2 | ≥ 100 | 100 |
